# Generation of High Peak Power Mode-Locked Green Pulses Based on WS_2_ and EOM: Experiment and Theory

**DOI:** 10.3390/molecules26154406

**Published:** 2021-07-21

**Authors:** Wenjing Tang, Wanggen Sun, Jing Wang, Kai Jiang, Wei Xia, Shengzhi Zhao

**Affiliations:** 1 School of Physics and Technology, University of Jinan, Jinan 250022, China; sps_tangwj@ujn.edu.cn (W.T.); 201921100081@mail.ujn.edu.cn (W.S.); ss_wangj@ujn.edu.cn (J.W.); sps_jiangk@ujn.edu.cn (K.J.); 2 School of Information Science and Engineering, Shandong University, Qingdao 266237, China

**Keywords:** solid-state, frequency modulated, mode-locked pulses, high peak power, rate equation

## Abstract

Based on an as-prepared high-quality WS_2_ film and an electro-optic modulator (EOM), a dual-loss-modulated low repetition rate mode-locking laser at 0.53 μm with high peak power is presented for the first time. The laser characteristics versus the pump power are investigated experimentally and theoretically. At a pump power of 10.67 W, the shortest pulse duration of 305 ps can be measured, corresponding to the highest peak power of 931 kW, which is much higher than those of the single passive modulated lasers with WS_2_-SA. A simple rate equation simulation was used to describe this dual-loss-modulated mode-locking green laser based on WS_2_ and EOM. The results of the numerical simulation are basically in accordance with the experimental values.

## 1. Introduction

Saturable absorbers (SAs) whose optical losses reduce as the optical intensity increases have become a critical component in pulsed lasers. For a substantial period of time, conventional SAs such as SESAMs were the main choice for lasers due to their flexibility and stability [1]. However, the drawbacks of a complex fabrication process and narrowband operating range make the application of conventional SAs limited. Subsequently, stimulated by the successful application of graphene and carbon nanotube [2,3], two-dimensional (2D) materials have quickly become the choice of SA for pulsed lasers. The relevant photonics applications of those 2D layered materials, such as black phosphorus (BP), topological insulators (TIs) and transition-metal dichalcogenides (TMDs), have also been investigated by researchers [4,5]. Among these 2D layered materials, TMDs are ideal choices for ultra-fast photonics, owing to their advantages of thickness-dependent properties and ultra-fast nonlinear optical property [6,7,8,9].

In the family of TMDs, WS_2_ as SA demonstrates better optical performance and mechanical stability than MoS_2_, MoSe_2_ and WSe_2_ [10]. Until now, monolayer or few-layer WS_2_ has been successfully applied as SAs for passively pulsed lasers from visible to mid-infrared wavelengths [10,11,12,13,14,15,16,17]. More importantly, WS_2_ as SA exhibits excellent mode-locking properties in all-solid-state lasers and fiber lasers because of its large thermal conductivity and high third order nonlinearity properties [14,18]. In comparison with other 2D SAs widely used for mode-locking, WS_2_ has the advantages of achieving large pulse energy and narrow pulse width [14,18,19]. However, there have been no related reports on the application of WS_2_-SA in solid-state lasers at 0.53 μm waveband. As we know, 0.53 μm green-beam sources with ultrashort pulse widths and high peak powers are especially attractive in many applications, such as pumping of Ti:sapphire lasers, processing of materials, generating fourth-harmonic of solid-state lasers and biomedical science [20,21,22,23]. To obtain an efficient and reliable green-beam source, the intracavity frequency-doubled technique is one of the most promising candidates. For this kind of frequency-doubled laser, large peak power can significantly improve the intracavity second harmonic (SH) conversion efficiency. However, because of the high repetition rate of continuous wave mode-locking and wide pulse duration of Q-switching, the aforementioned single passive lasers were difficult to obtain high peak power. New methods for reducing repetition rate and compressing pulse width are highly demanded.

In comparison with single modulation methods, Q-switching and mode-locking (QML) technique under a dual-loss modulation mechanism can realize laser output with short pulse duration and large pulse energy simultaneously [24]. In this paper, by employing WS_2_-SA and an electro-optic modulator (EOM) as the dual-loss modulator, we firstly realized the output of frequency-adjustable high peak power subnanosecond green pulses from a diode-pumped QML Nd:Lu_0.15_Y_0.85_VO_4_/KTP laser. Here, mixed crystal Nd:Lu_0.15_Y_0.85_VO_4_ is used as the gain medium for its excellent optical properties [25,26]. Based on the dual-loss modulation mechanism, the pulse width of the Q-switched envelope can be greatly compressed until only one mode-locking pulse is left. At the pump power of 10.67 W, the shortest pulse width of 305 ps was obtained, corresponding to a peak power of 931 kW. Additionally, the theoretical investigation using the rate equation theory on the laser operating characteristics was demonstrated in this paper. Simple coupled rate equations for the dual-loss-modulated mode-locking green laser are introduced and solved numerically. The numerical solutions of the equations are basically in accordance with the experimental results, which can also provide the guidance for the realization of the experiment.

## 2. WS_2_ Preparation and Characterization

Using the ultrasonic-assisted vertical evaporation method, few-layer WS_2_ nanosheets were fabricated and characterized. Firstly, 20 mg WS_2_ powder was added to SDS (sodium dodecyl sulfate) aqueous solution with the concentration of 0.1%. Secondly, the aqueous solution was ultrasonically agitated for 12 h and centrifuged to remove large WS_2_ clusters. Then, the upper layer of the centrifuged solution with high absorption was obtained. Thirdly, diluted the solution and poured it into a polystyrene cell. A hydrophilic quartz substrate was inserted vertically into the cell and put the polystyrene cell at the atmosphere for gradual evaporation until the WS_2_ nanosheets adhered to the quartz surface. Finally, a high-quality few-layer WS_2_ sample can be obtained.

**Figure 1:**
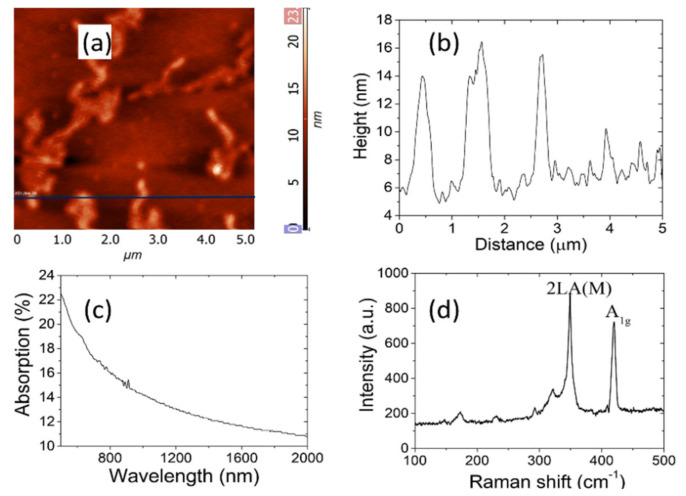
(**a**) AFM image; (**b**) corresponding height profiles; (**c**) absorption spectrum; (**d**) Raman spectra.

The characterizations of the few-layer WS_2_ sample are summarized in Figure 1. The atomic force microscopy (AFM) image (Figure 1a) of the sample shows the WS_2_ flakes structure. One can see that the nanosheets spread evenly over the hydrophilic quartz substrate. The thickness curve of WS_2_ nanosheets is shown in Figure 1b. An average thickness of 7–9 nm was obtained, indicating that the layer number of the WS_2_ sample was 8–10. The absorption spectrum of the WS_2_ film was measured by a Hitachi U 4100 spectrophotometer and shown in Figure 1c, which proves that the WS_2_ SA possess broadband absorption characteristics. Further Raman Characterization in Figure 1d shows that the E_2g_ Raman peak (an in-plane motion) and A_1g_ Raman peak (out-of-plane motion) located at 349.2 cm^−1^ and 419.7 cm^−1^, respectively. The intensity ratio between the E_2g_ peaks and the A_1g_ peaks indicates that the few-layer structure of WS_2_ film has been successfully fabricated [27].

**Figure 2:**
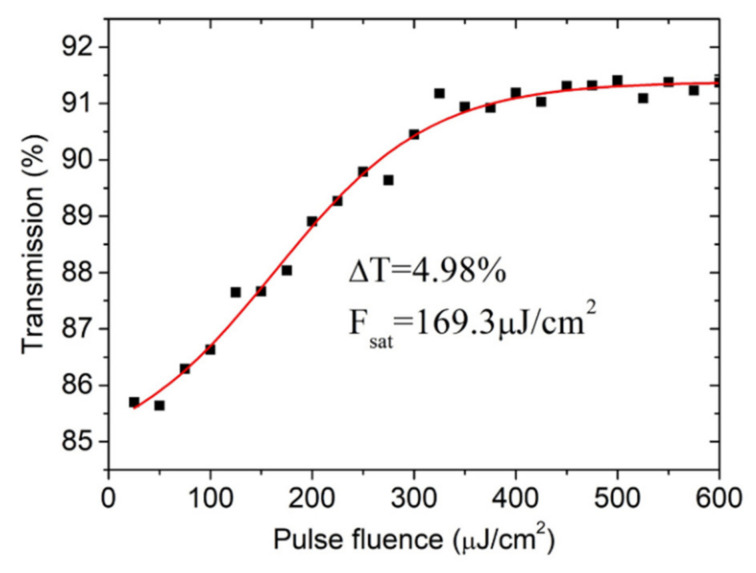
Nonlinear transmittance curve of the WS_2_-SA versus the input pulse fluence.

To further confirm the saturable absorption capability of the WS_2_-SA sample, we designed a balanced twin-detector measurement system to investigate the nonlinear optical absorption characteristics of the WS_2_-SA. The laser source is a homemade picosecond mode-locking laser at 1.06 μm. Figure 2 shows the obtained nonlinear transmission curve of the WS_2_ sample. The modulation depth and the saturation intensity were determined to be about 4.98% and 169.3 μJ/cm^2^, respectively.

## 3. Experimental Setup and Results

### 3.1. Experimental Setup

**Figure 3:**
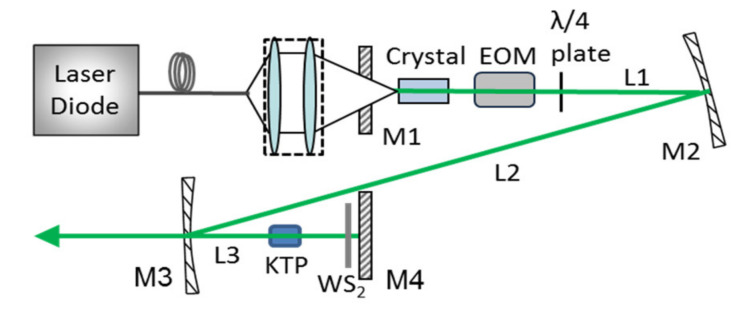
Schematic diagram of the dual-loss modulation Nd:Lu_0.15_Y_0.85_VO_4_/KTP green laser.

The schematic configuration of the dual-loss modulation Nd:Lu_0.15_Y_0.85_VO_4_/KTP green laser with EO and WS_2_-SA is shown in Figure 3. The pump source is a commercial fiber coupled Laser diode operating at 808 nm. By an optical coupled system, the pump beam with a spot radius of 200 μm is collimated and focused into the laser gain medium. To obtain stable and efficient QML pulse, a four-mirror-Z-fold-cavity is designed with three cavity arms lengths of 57, 76 and 9 cm, respectively. M1 as a flat mirror, anti-reflection (AR) coated at 808 nm on both surface and high-reflection (HR) coated at 1064 and 532 nm on the inside surface, is adopted as the input mirror. The resonator mirror M2 is a concave mirror with a radius of curvature (ROC) of 500 mm. The output mirror M3 with a ROC of 150 mm is HR coated at 1064 nm and AR coated at 532 nm. Another resonator mirror, M4, is a flat mirror, the same as M2, and both are HR coated at 1064 nm and 532 nm. The laser gain medium is an a-cut mixed crystal Nd:Lu_0.15_Y_0.85_VO_4_, which are AR coated at 808 and 1064 nm on both surfaces. The frequency-doubling crystal KTP, AR coated at 1064 and 532 nm on both facets, is cut for type-II phase matching at 1064 nm. A thermo-electric cooler is used to dissipate the heat deposition in the laser crystal and KTP. With a λ/4 plate, an EOM (BBO crystal) is employed as the active modulator. As we know, BBO crystal is beneficial to compress the pulse duration because of its fast switching and excellent hold-off ability. The home-made WS_2_ film is used as the SA. A PM100D Energy/Power Meter (Thorlabs Inc., Newton, NJ, USA) was used to measure the output power. A 16 G digital oscilloscope (Agilent DSO-X91604A, 80 G samples/s sampling rate, Keysight Technologies Inc., Santa Rosa, CA, USA) and a fast pin photodiode detector with the response time of 14 ps (New Focus 1414, Newport Co., Irvine, CA, USA) is used to record the pulse temporal behavior.

### 3.2. Experimental Results and Discussion

For this doubly modulated green laser system with WS_2_-SA and EOM, there are two operating stages, i.e., the QML pulse generation stage and the mode-locking pulse generation stage. Stable QML pulses were measured at a low pump power, in which EOM controlled the repetition rate of the Q-switched envelopes while the cavity roundtrip time determined the repetition frequency of the mode-locking pulses underneath the Q-switched envelopes. With the increase of pump power, the inversion population density in Nd:Lu_0.15_Y_0.85_VO_4_ crystal can be greatly improved and the pulse widths of Q-switched envelopes can be gradually compressed. When the pump power is large enough, the Q-switched envelope can be compressed to contain only one mode-locked pulse with sub-nanosecond pulse duration. Then the laser enters the single mode-locking stage. A mode-locked pulse train with the repetition rate equal to the modulated frequency of EOM can be generated.

**Figure 4:**
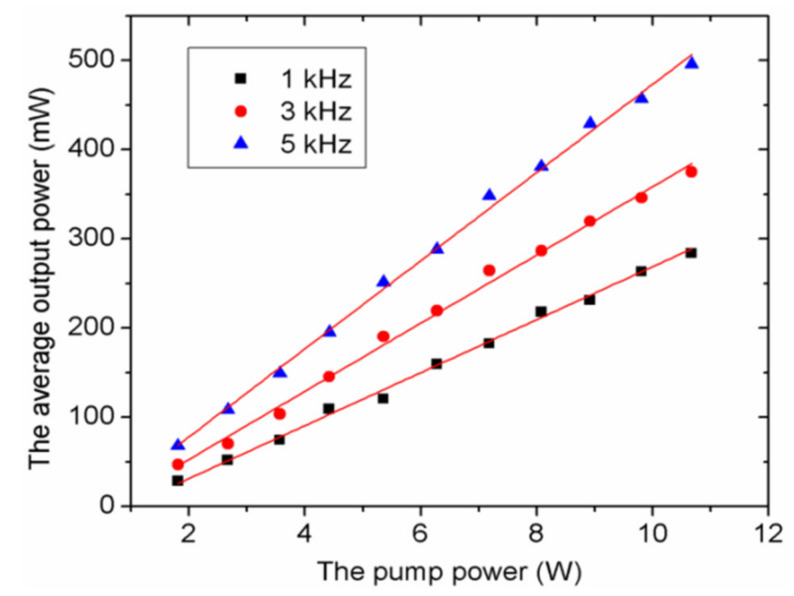
Dependence of the average output power on the pump power at different modulation frequencies. Symbols, experimental data; solid curves, fitted curves.

In the experiment, the pulse characteristics of the dual-loss-modulated green laser were investigated and summarized. Figure 4 shows the average output powers versus the pump power for different modulation frequencies. One can see that the average output powers increase linearly with the increase of the pump power and the modulated frequency. At a pump power of 10.67 W, the maximum average output powers of 284, 375, and 496 mW under 1, 3, and 5 kHz were obtained, respectively.

**Figure 5:**
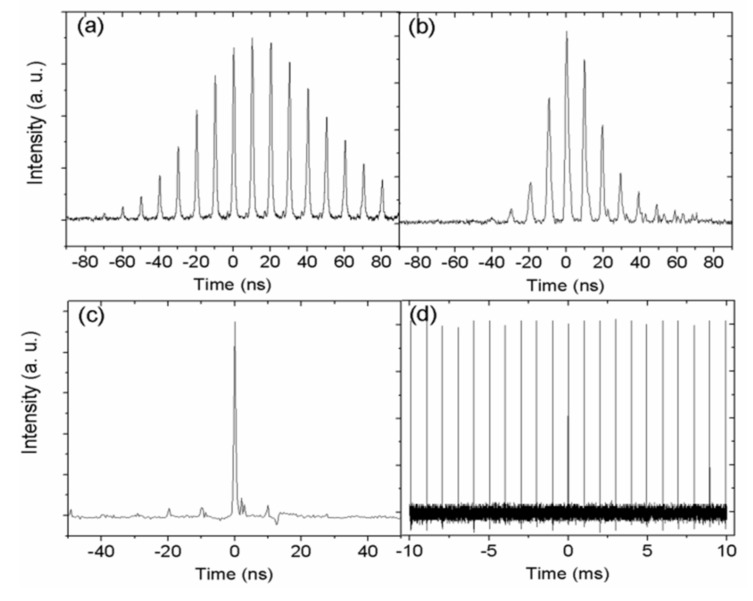
Pulse shapes of the dual-loss-modulated QML green laser at different pump powers with 1 kHz repetition rate: (**a**) 1.81 W; (**b**) 3.57 W; (**c**) 5.36 W; (**d**) 10.67 W.

To show the variation of the pulse envelope more intuitively, the temporal pulse profiles at a 1 kHz repetition rate of EOM are recorded and shown in Figure 5. Obviously, the number of mode-locked pulses underneath a Q-switched envelope decreases monotonically with increasing pump power. With pump powers of 1.81 and 3.57 W, there are eight and four mode-locked pulses underneath a Q-switched envelope, respectively. When the pump power reaches 5.36 W, only one mode-locking pulse exists under an envelope. This is mainly caused by the choice of active Q-switching. The loss of other pulses in the envelope is higher than the gain, and it is hard to realize the amplification of stimulated emission. Nevertheless, we can still observe the appearance of adjacent pulses with the 2L/C interval in Figure 5c. Figure 5d exhibits a single mode-locking pulse train obtained at 1 kHz under 10.67 W pump power. The pulse-to-pulse amplitude instability is less than 4%, which is much smaller than that of the singly passively Q-switched laser with WS_2_-SA. In addition, it is worth emphasizing the high optical damage threshold of the WS_2_ film. During the whole experiment, no optical damage of the WS_2_-SA was observed.

**Figure 6:**
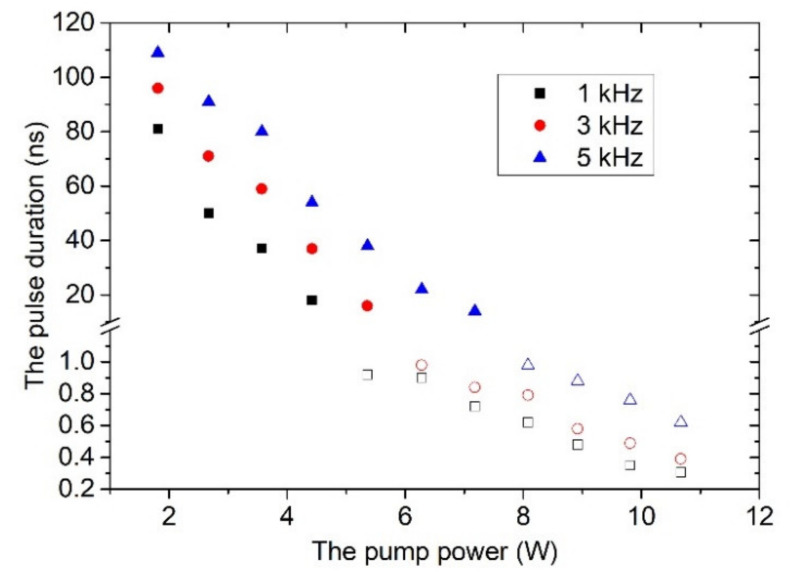
Dependence of the pulse widths versus the pump power. Solid symbols: the QML stage; open symbols: the single mode-locking stage.

**Figure 7:**
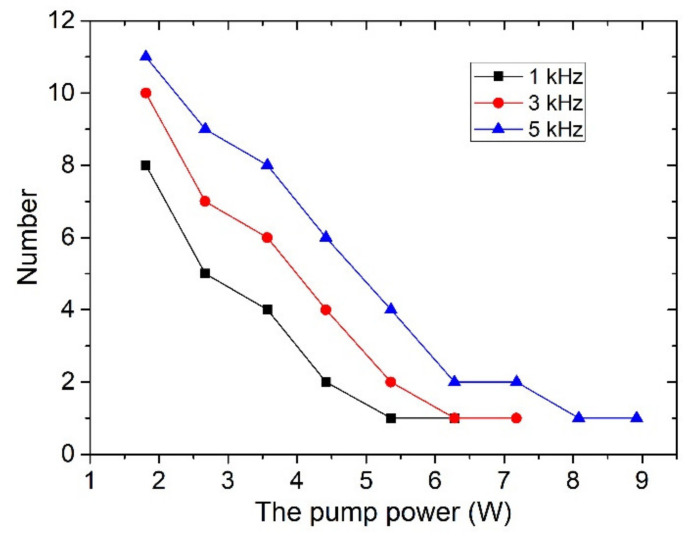
The number of mode-locking pulses covered by one envelope versus the pump power.

The pulse durations versus the pump power for different modulation frequencies are described in Figure 6. At the QML stage, the number of mode-locking pulses covered by one envelope is also shown in Figure 7. One can see that the pulse durations of Q-switched envelopes given as solid symbols decrease rapidly with the increase of pump power. With increasing pump power, the laser enters the single mode-locking stage. The pump powers for generating sub-nanosecond single mode-locking pulses are called threshold pump powers. Threshold pump powers of 5.36, 6.28 and 8.08 W were obtained for the modulated rates of 1, 3 and 5 kHz, respectively. Evidently, a low modulation rate is beneficial for the compression of the pulse durations. At this stage, the pulse durations of single mode-locking pulses given as open symbols is still decreasing with increase of the pump power. At a pump power of 10.67 W, the shortest pulse durations of 305, 390 and 625 ps were obtained at 1, 3 and 5 kHz, respectively.

**Figure 8:**
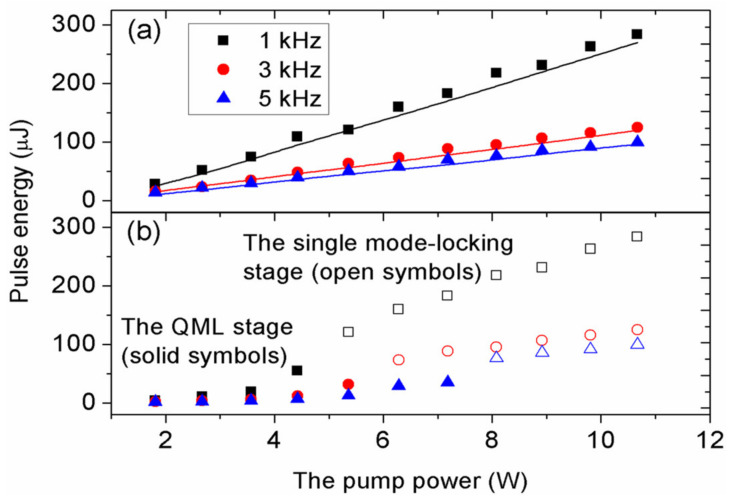
(**a**) Pulse energy of Q-switched envelope and (**b**) pulse energy of mode-locking pulses versus the pump power; symbols, experimental data; solid curves, theoretical results.

According to the average output powers and the modulated frequencies, the pulse energies of the Q-switched envelope were calculated and are presented in Figure 8a. Although a higher repetition rate can generate a higher average output power, it has no advantage in terms of obtaining a larger pulse energy. The obtained maximum pulse energies were 284, 125, and 99.2 μJ for the modulation frequencies of 1, 3, and 5 kHz at the pump power of 10.67 W. As we know, at the QML stage, there are multiple mode-locked pulses in a Q-switched envelope. To investigate the variation of mode-locking pulse energies, the pulse energy of single Q-switched envelope is divided by the number of mode-locking pulses, and then the average mode-locking pulse energy can be obtained, as shown in Figure 8b. The mode-locking pulse energies obtained at the QML stage (solid symbols) are much smaller than the Q-switched envelope energies. However, at the mode-locking pulse generation stage, almost all the pulse energy of the Q-switched envelope is concentrated into the single mode-locking pulse. The pulse energy of the Q-switched envelope can be regarded as equal to that of the single mode-locking pulse (open symbols). Thus, it can be concluded that the dual-loss modulation technique is an excellent choice in enhancing the pulse energy.

**Figure 9:**
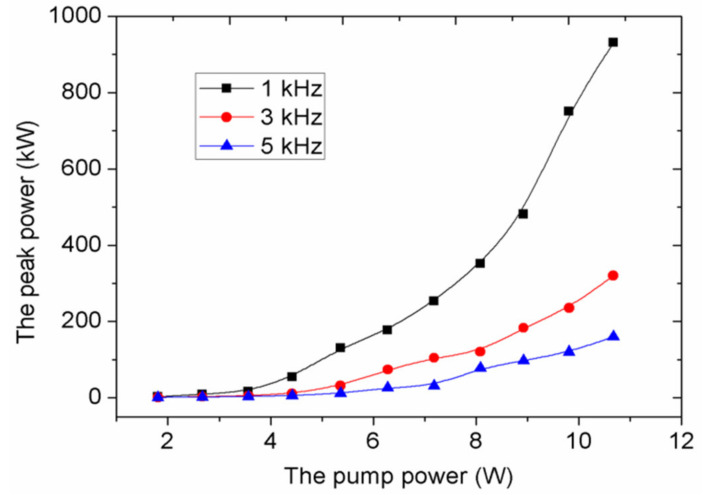
Peak power of mode-locking pulses versus the pump power for different modulation frequencies.

According to the pulse energy and pulse width, the mode-locking pulse peak powers can be calculated, as exhibited in Figure 9. We can find that a low modulated frequency has obvious advantage in generating large peak power. In particular, at 1 kHz, the peak power has a tendency of growing exponentially as the increase of the pump power. At the pump power of 10.67 W, the maximum peak powers are 931, 320 and 160 kW for 1, 3 and 5 kHz, respectively. To our knowledge, the pulse energy and the pulse peak power of this dual-loss modulation mode-locking green laser is much higher than those generated by other single modulated pulsed lasers based on WS_2_-SA reported previously [10,11,15,16,28,29,30,31].

## 4. Theoretical Analysis and Numerical Simulation

A simple mathematical model of the dual-loss modulation QML laser was developed on the basis of the rate equation theory and the fluctuation mechanism [32,33,34,35]. Considering the Gaussian spatial distribution approximation, the intracavity photon density $\phi \left(r,t\right)$ of the fundamental wave for the TEM_00_ mode can be expressed as [32]:

(1) $$\phi \left(r,t\right)=\underset{k=0}{\sum }\Phi_{k}f\left(t-t_{k}\right)\exp\left(-\frac{2r^{2}}{\omega_{l}^{2}}\right)$$

where $\omega_{l}$ is the average radius of the TEM_00_ mode oscillating laser in the cavity; *r* is the radial coordinate and *t* is the time; $\Phi_{k}$ is the relative amplitude of the mode-locking pulses at the *k_th_* roundtrip; $\;t_{k}=kt_{r}$ and $f\left(t\right)=sech^{2}\left(t/\tau_{p}\right)/2\sigma_{m}c\tau_{p}$ describes the mode-locking pulse evolving from the noise, where $\sigma_{m}$ is the stimulated emission section of the gain medium. Here, $t_{r}=2\left[nl_{a}+n_{1}l_{E}+n_{2}l_{s}+n_{3}l_{kp}+\left(L_{c}-l_{a}-l_{E}-l_{s}-l_{kp}\right)\right]/c$ represents the roundtrip time, $n,n_{1},\;n_{2}$ and $n_{3}$ are the refractive indices of the gain medium, EOM, WS_2_-SA and KTP crystal, respectively; $l_{a},\;l_{E},\;\;l_{s}$ and $l_{kp}$ are the lengths of the gain medium, EO modulator, WS_2_-SA and KTP crystal, respectively (here,$\;l_{s}\approx 0$); $\tau_{p}\;$is related to the FWHM mode-locking pulse duration at the fundamental wavelength by$\;\tau =1.76\tau_{p}$.

The relevant energy levels of Nd^3+^ ions in Nd:Lu_0.15_Y_0.85_VO_4_ crystal which can be depicted by a four-level model and all up-conversion processes can be neglected [36]. Furthermore, because the mode-locked pulse shape is stable after several roundtrips, the mode-locked pulse duration can be set as a fixed value. By considering the saturated absorption of WS_2_, the modulation loss of EOM and the nonlinear loss of KTP at the same time, the coupled rate equations can be expressed as [37,38]:

(2) $$\Phi_{k}=\Phi_{k-1}exp\left\{\frac{2}{\pi \omega_{l}^{2}}\int_{0}^{\infty }\left\{2\sigma_{m}n\left(r,t_{k}\right)l_{a}\frac{\omega_{l}^{2}}{\omega_{m}^{2}}\exp\left(-\frac{2r^{2}}{\omega_{m}^{2}}\right)-\delta_{s}\frac{\omega_{l}^{2}}{\omega_{A}^{2}}\exp\left(-\frac{2r^{2}}{\omega_{A}^{2}}\right)-\delta_{e}\frac{\omega_{l}^{2}}{\omega_{E}^{2}}\exp\left(-\frac{2r^{2}}{\omega_{E}^{2}}\right)-\delta_{kp}\frac{\omega_{l}^{2}}{\omega_{kp}^{2}}\exp\left(-\frac{2r^{2}}{\omega_{kp}^{2}}\right)-Lexp\left(-\frac{2r^{2}}{\omega_{l}^{2}}\right)\right\}2\pi rdr\right\}$$

(3) $$n\left(r,t_{k}\right)=\exp\left(-\frac{t_{k}}{\tau_{m}}\right)\overset{k-1}{\underset{m=0}{\prod }}\exp\left[-\frac{\omega_{l}^{2}}{\omega_{m}^{2}}\exp\left(-\frac{2r^{2}}{\omega_{m}^{2}}\right)\Phi_{m}\right]\left\{R_{in}\left(r\right)\times \exp\left(\frac{t_{k}}{\tau_{m}}\right)\int_{0}^{t_{k}}\overset{k-1}{\underset{m=0}{\prod }}\exp\left[\frac{\omega_{l}^{2}}{\omega_{m}^{2}}\exp\left(-\frac{2r^{2}}{\omega_{m}^{2}}\right)\Phi_{m}\right]dt+n_{i}exp\left(-\frac{2r^{2}}{\omega_{m}^{2}}\right)\right\}.\;$$

where$\;\omega_{i}\left(i=m,A,E\right)$ is the average beam radius of TEM_00_ mode at the position of the gain medium, the WS_2_-SA and the EOM, respectively, which can be calculated by the ABCD matrix theory. $n\left(r,t_{k}\right)$ is the average population-inversion density at the kth roundtrip. The pump rate can be expressed as

(4) $$R_{in}\left(r\right)=\frac{P_{in}\exp\left(-\frac{2r^{2}}{\omega_{p}^{2}}\right)\left[1-\exp\left(-\alpha l_{a}\right)\right]}{hv_{p}\pi \omega_{p}^{2}l}$$

where $P_{in}$ is the pump power,$\;hv_{p}$ is the single photon energy of the pump light,$\;\omega_{p}$ is the average radius of the pump beam, α is the absorption coefficient of the gain medium. $\tau_{m}$ represents the lifetime of the upper laser level. The saturable and non-saturable absorption losses of WS_2_ can be expressed as

(5) $$\delta_{s}=\alpha_{ns}+\frac{\alpha_{s}I_{sat}}{I_{sat}-\frac{\gamma hv_{g}\omega_{l}^{2}}{2\sigma_{m}\tau_{p}\omega_{A}^{2}}\exp\left(-\frac{2r^{2}}{\omega_{l}^{2}}\right)\Phi_{k}sech^{2}\left(\frac{t}{\tau_{p}}\right)}$$

where $\alpha_{s}$ and $\alpha_{ns}\;$are the saturable and non-saturable absorption of WS_2_-SA, respectively, which can be obtained from Figure 1b. $hv_{g}$ is the single photon energy of the lasing emissions. $\gamma =A_{g}/A_{s}$, where $A_{g}$ and $A_{s}$ are the lasing beam area in the laser crystal and the WS_2_-SA, respectively. EOM as an active modulator is a fast speed switcher with short turnoff time. For a doubly QML laser with EOM and WS_2_-SA, the pulse width of the Q-switched envelope is much wider than the turnoff time of EOM. Therefore, in the theoretical simulation, the turnoff time of EOM can be neglected. As soon as the switcher is turned on, all losses of EOM in the cavity can be represented by the average loss $\delta_{e}$. Additionally, for this frequency-doubled laser, the SH conversion is generally regarded as the nonlinear loss of the fundamental wave [37]. $\delta_{kp}$ is the total nonlinear loss of the KTP crystal. Under the small signal approximation, the nonlinear loss $\delta_{kp}$ at the *k_th_* roundtrip can be deduced as:

(6) $$\delta_{kp}\left(r,t\right)=\frac{hvK_{N}}{2\sigma \tau_{p}}\frac{\omega_{l}^{2}}{\omega_{kp}^{2}}\exp\left(-\frac{2r^{2}}{\omega_{kp}^{2}}\right)\Phi_{k}sech^{2}\left(\frac{t}{\tau_{p}}\right)$$

where $\omega_{kp}$ is the average beam radius of TEM_00_ mode at the position of KTP crystal. $K_{N}=\omega^{2}d_{\mathrm{eff}}^{2}l_{kp}^{2}/c^{3}\varepsilon_{0}n_{e}^{2\omega }n_{o}^{\omega }n_{e}^{\omega }$, ω is the angle frequency of the fundamental wave,$\;d_{\mathrm{eff}}$ is the effective nonlinear coefficient, $\varepsilon_{0}$ is the dielectric permeability of vacuum; $n_{e}^{2\omega }$,$\;n_{o}^{\omega }$ and $n_{e}^{\omega }$ are the SH and fundamental wave refractive indices. In addition, L  represents passive losses for double cavity path.

According to Equations (1)–(6) the output second-harmonic power couple out of the cavity can be expressed as [37,38]:

(7) $$P_{2\omega }\left(t\right)=\frac{A_{p}hv_{g}}{8\sigma_{m}\tau_{p}}K_{N}\frac{\omega_{l}^{4}}{\omega_{kp}^{4}}\underset{k=0}{\sum }\Phi_{k}^{2}sech^{4}\left(\frac{t-t_{k}}{\tau_{p}}\right)$$

**Figure 10:**
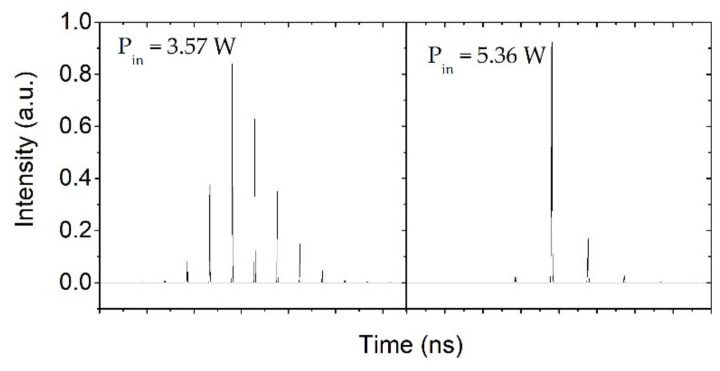
Numerically simulated pulse shapes at 1 kHz repetition rate.

The values of relevant parameters are presented in Table 1. Using these parameters, for a given initial value of $\Phi_{0}$, by numerically solving Equations (1)–(7), we can obtain $\Phi_{k}$. Meanwhile, the output second-harmonic power couple and the total output pulse energy of the Q-switched envelope out of the cavity can be obtained. Figure 8a shows the results of the numerical simulation of the Q-switched envelope pulse energy with solid curves, which were basically consistent with the experimental results. Additionally, based on the above equations, the temporal shapes of the dual-loss modulation QML pulses were numerically simulated and are shown in Figure 10. At a pump power of 5.36 W, the simulation results we obtained almost achieved a single mode-locked pulse output, which is basically in accordance with the experimental results shown in Figure 5.

## 5. Conclusions

In conclusion, by simultaneously employing EOM and WS_2_-SA, a frequency-adjustable high peak power Nd:Lu_0.15_Y_0.85_VO_4_/KTP green laser is demonstrated for the first time. In comparison with other singly mode-locked or Q-switched lasers based on WS_2_-SA reported previously, the peak power obtained from the dual-loss modulation green laser is the largest one. The rate equation theory was also used to further analyze the experimental results. The results show this dual-loss modulation system can satisfy the requirements of lasers with high stability, specific low repetition rate as well as high peak power.

## Figures and Tables

**Table 1:** Parameters of the theoretical calculation ^1^.

Symbol	Values	Ref.	Symbol	Values
σ_m_	8.7 × 10^−19^ cm^2^	[39]	l_E_	50 mm
τ_m_	124 μs	[39]	ω_A_	96 μm
α	5.32 cm^−1^	[39]	ω_p_	200 μm
n	2.045	[40]	ω_m_	300 μm
n_1_	1.65	[40]	ω_E_	121 μm
δ_e_	0.1	[40]	ω_kp_	106 μm
c	3 × 10^8^ m/s	[40]	ω_l_	166 μm
L	0.07		α_s_	0.0498
$\tau_{p}$	200 ps		α_ns_	0.092
l_a_	10 mm		d	8 mm

^1^ Parameters without references were all measured in this work.

## Data Availability

Not applicable.
